# Individual-based modelling of population growth and diffusion in discrete time

**DOI:** 10.1371/journal.pone.0176101

**Published:** 2017-04-20

**Authors:** Natalie Tkachenko, John D. Weissmann, Wesley P. Petersen, George Lake, Christoph P. E. Zollikofer, Simone Callegari

**Affiliations:** 1 Institute for Computational Science, University of Zurich, Zurich, Switzerland; 2 Department of Anthropology, University of Zurich, Zurich, Switzerland; 3 Seminar for Applied Mathematics, ETH Zurich, Zurich, Switzerland; University of Bristol, UNITED KINGDOM

## Abstract

Individual-based models (IBMs) of human populations capture spatio-temporal dynamics using rules that govern the birth, behavior, and death of individuals. We explore a stochastic IBM of logistic growth-diffusion with constant time steps and independent, simultaneous actions of birth, death, and movement that approaches the Fisher-Kolmogorov model in the continuum limit. This model is well-suited to parallelization on high-performance computers. We explore its emergent properties with analytical approximations and numerical simulations in parameter ranges relevant to human population dynamics and ecology, and reproduce continuous-time results in the limit of small transition probabilities. Our model prediction indicates that the population density and dispersal speed are affected by fluctuations in the number of individuals. The discrete-time model displays novel properties owing to the binomial character of the fluctuations: in certain regimes of the growth model, a decrease in time step size drives the system away from the continuum limit. These effects are especially important at local population sizes of <50 individuals, which largely correspond to group sizes of hunter-gatherers. As an application scenario, we model the late Pleistocene dispersal of *Homo sapiens* into the Americas, and discuss the agreement of model-based estimates of first-arrival dates with archaeological dates in dependence of IBM model parameter settings.

## Introduction

There is an increasing interest in individual-based models (IBMs) in ecology and anthropology [1]. These are spatio-temporal dynamical models of individuals [1–4] acting by biology-inspired rules [5–7]. An individual can be a plant or an animal or even a group. The system dynamics arise bottom-up via the nonlinear interactions among individuals. These models capture inter-individual variation and stochasticity that are omitted in continuous, deterministic models [5]. Stochasticity plays a critical role in population dynamics as a smaller population has stronger fluctuations in the number of individuals that may lead to extinction [8–10]. IBMs have been applied to spatial patch population dynamics [2, 11], spatially expanding populations [12–14], and epidemics [15].

IBMs are different from stochastic population-based simulations where individual actions such as reproduction, death, movement etc. are not taken separately by each entity, but are determined at once for the whole population [16, 17]. An IBM may have a deterministic continuum limit, i.e., the model can be described with deterministic equations in the limit of a high number of individuals and small time steps, and therefore be suitable to explore a wide range of regimes from discrete and stochastic to continuous and deterministic.

Analytical and numerical studies of the birth-death process (e.g. logistic growth) and Fisher-Kolmogorov reaction-diffusion population models [18–20] have predicted that discreteness and stochastic fluctuations have a strong impact on the large-scale population dynamics. The emergent properties of the system, such as the propagation speed of the population or the time-to-extinction, diverge from the properties of the continuous equations. Renshaw studied the stochastic birth-death process analytically as a one-step Markov process with continuous time [21]. He found that the average number of individuals in the steady-state is lower than in the deterministic case, and that the stochastic system approaches deterministic predictions when the fluctuations decrease. The propagation speed *v* in stochastic reaction-diffusion systems is lower than for the deterministic traveling wave [22–27]. Several studies [22, 23, 28] explained the slowdown of the wave with the processes on its tip and found an analytical approximation of *v* up to the second order. This approximation was confirmed in a study of fitness waves in microbial evolution [25]. Traveling waves in the strong-noise limit were studied in [24, 26], where wave fronts composed of a few rugged kinks instead of smooth sigmoidal profiles were observed.

Individual-based growth and growth-diffusion models are often implemented with continuous-time-formulations, where the time step is dynamically chosen to ensure that individual “actions” happen one at a time [2, 29, 30]. In computational terms, if actions of individuals depend on each other they cannot be executed in parallel. Thus, the continuous-time approach scales poorly to large populations.

Here we describe a discrete-time implementation of growth and diffusion that scales to large spatial domains, large numbers of individuals, and long time spans [31]. At each fixed time step, all individuals act simultaneously and independently. Due to the discrete-time implementation, the model is amenable to parallelization: calculations can be split among processors with each working on a fraction of the individuals. Individuals decide on three basic “actions”—deaths, births, and movements, and all may perform them.

We first analyze the movement and the growth terms separately, then present the full Fisher-Kolmogorov growth-diffusion IBM. Numerical simulations are used to study the stochasticity and the large-scale properties using parameters relevant for the spatio-temporal dynamics of hunter-gatherer populations. The major aim here is to investigate how the outcome of an IBM-based implementation of growth and dispersal deviates form the expected properties of the outcome of continuum models. As an example, we simulate the dispersal of modern humans in the Americas during the late Pleistocene.

## Description of the model

The Fisher-Kolmogorov (FK) equation models spatial dispersal and local change via diffusion and growth terms [32].

We now introduce the discrete-time models for the diffusion and growth terms, indicating that their continuum limits are the diffusion and the logistic growth equations. Combining the two terms yields a discrete-time stochastic version of the Fisher-Kolmogorov model. Simulations are performed with the QHG code—an agent-based modeling framework written in C++ that uses parallel computing [31]. Some simulations of the growth term, for which high performance was not needed, are performed using a Python implementation of the growth model. The QHG code, Python script, and scripts for the analysis are available on https://github.com/uzh/QHG.

### Diffusion term

In the one-dimensional case, the deterministic diffusion equation is

$$\begin{array}{c} \frac{\partial \rho (x,t)}{\partial t}=D\frac{\partial^{2}\rho \left(x,t\right)}{\partial x^{2}} \end{array}$$ (1)

where *ρ*(*x*, *t*) is the population density at time *t* and position *x*, and *D* is a constant diffusion coefficient (see e.g. [32]). Assuming the population starts in a localized region of space, the second moment of positions for large *t* is proportional to time:

$$\begin{array}{c} \text{Var}(x)=2Dt\;. \end{array}$$ (2)

In our IBM, the diffusion process is modeled at the individual level via an isotropic random walk on a Cartesian grid [32]. Each grid cell has a fixed number of neighbors: 2 in the one-dimensional and 4 in the two-dimensional case. Each individual moves with probability *P*_move_ to a neighboring cell at a given time step. Movements are synchronous and independent within a time step *τ*, and thus the IBM is a discrete-time model [8].

This is in contrast with the so-called “continuous-time model”, where the timing of individual actions is based on a Poisson process so that only one action occurs at a time [8].

Our model is a binomial process, with success probability *P*_move_, and sample size equal to the total number of individuals *N*_0_. When *N*_0_ *P*_move_ → 0 (or equivalently *τ* → 0), our model approaches the continuous-time process.

The large-scale diffusion coefficient is calculated using the microscopic parameters of a random walk (see also [32]):

$$\begin{array}{c} D=P_{\text{move}}\left[P_{\text{direction}}\frac{\Delta x^{2}}{\tau }\right]. \end{array}$$ (3)

*P*_direction_ is the probability to move to a specific neighbor of the current cell, Δ*x* is equal to the distance between two neighboring cells (the length of the spatial step), and *τ* is the length of the time step; here Δ*x* = 1 and *τ* = 1. For isotropic movement, *P*_direction_ = 1/*n*_neighbors_.

S1 Appendix predicts that in the limit of decreasing spatial and temporal steps, the model approaches the diffusion Eq (1).

### Growth term

A widely used deterministic model for density-dependent population dynamics is the logistic growth equation

$$\begin{array}{c} \frac{\text{d}\rho }{\text{d}t}=r\rho \left(1-\frac{\rho }{K}\right). \end{array}$$ (4)

*ρ* is the population density, *r* is the population growth rate, and *K* is the equilibrium density, the so-called carrying capacity [32] owing to limited resources in a spatial region.

**Fig 1:**
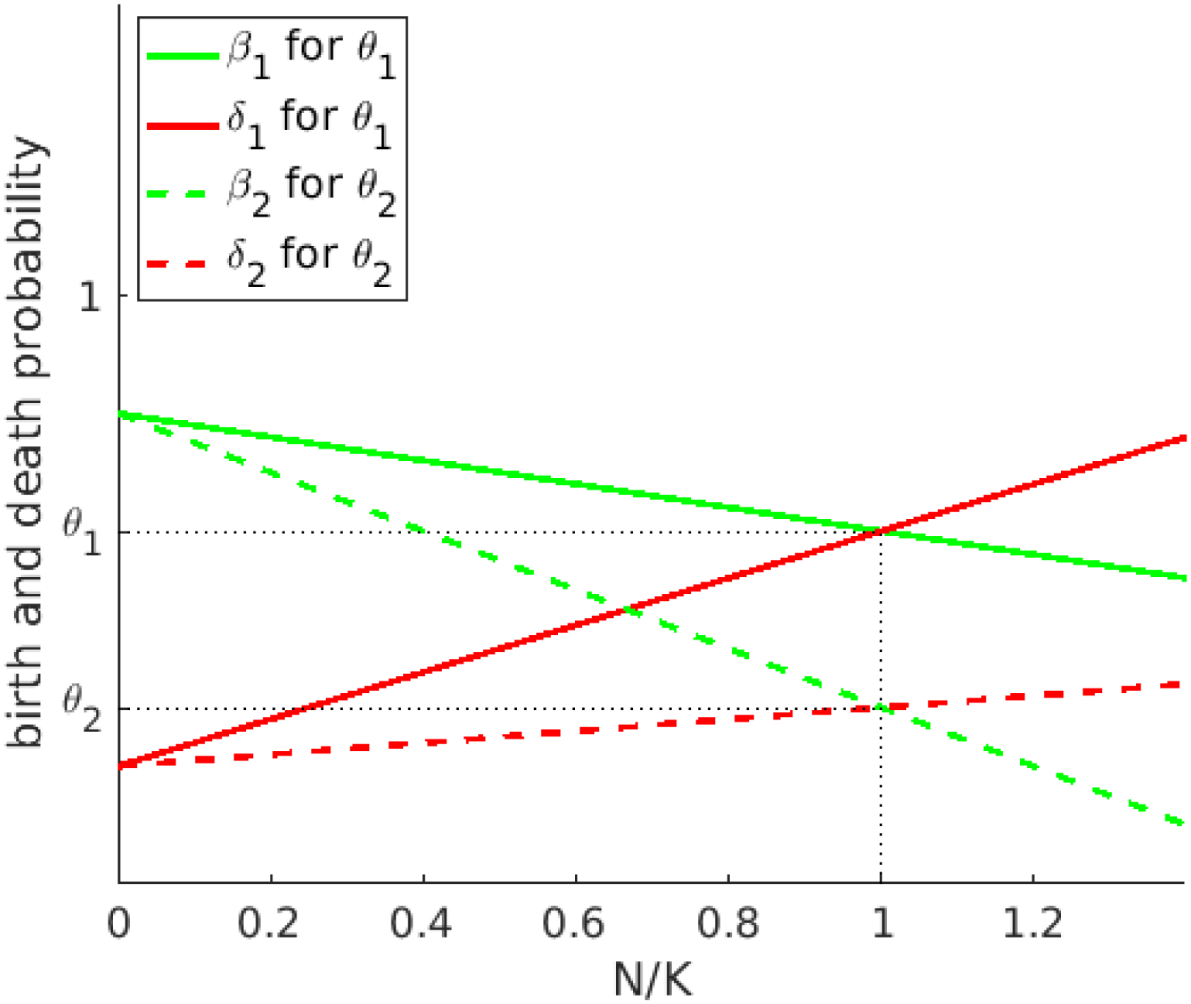
The dependency of the birth *β* (Eq (5)) and the death probability *δ* (Eq (6)) on normalized local population density *N*/*K*. Two different turnovers are considered: *θ*_1_ and *θ*_2_. When the number of individuals equals the carrying capacity *N*/*K* = 1, the equation *β* = *δ* = *θ* is fulfilled. Thus, as shown in the figure, the same carrying capacity can be reached with different turnovers [16].

There are many ways to define a stochastic model where the zero-noise limit approaches the logistic model (Eq (4)) [2, 21]. In our IBM, each individual can spawn (give birth to) a new one, or die, with probabilities *β* and *δ*, respectively. These reproduction and death behaviors depend on the local population density in the individual’s cell as follows:

$$\begin{array}{c} \beta =b_{0}-\left(b_{0}-\theta \right)N/K \end{array}$$ (5)

$$\begin{array}{c} \delta =d_{0}+\left(\theta -d_{0}\right)N/K\;, \end{array}$$ (6)

where *b*_0_ and *d*_0_ are the small-population limits of birth and death probabilities, respectively [16] (see Fig 1). *N* is the current number of individuals in the given cell. The deterministic population growth rate is *r* = (*b*_0_ − *d*_0_)/*τ*.

An additional parameter is the turnover *θ*, which is equal to the birth and death probabilities when the local population reaches its carrying capacity (*β* = *δ* = *θ* for *N* = *K*). Thus, *θ* defines the expected fraction of individuals that will be born and die at each time step when the cell’s population is *K*. In the model, *θ* is bounded in two directions: *d*_0_ < *θ* < *b*_0_, with *d*_0_ < *b*_0_. Note that *θ* only appears in the stochastic formulation and has no equivalent in the classical deterministic model. The same carrying capacity can be associated with different turnovers, and all models with same *K*, *b*_0_, and *d*_0_ converge to the same continuous model. To summarize, *β*, *δ* are interpreted as probabilities in the stochastic model, i.e., the parameters and the resulting growth rate *r* range from 0 to 1 by appropriate rescaling of the time unit *τ*.

**Fig 2:**
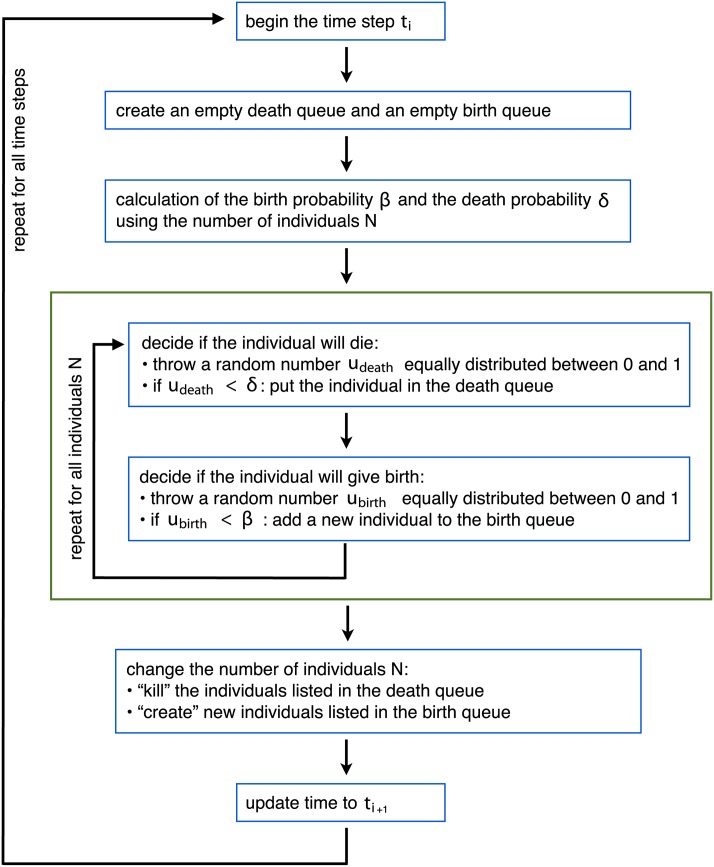
Growth model: Workflow during one time step *t*_*i*_. At each time step, the expected number of newborn and of dying individuals is *βN* and *δN*, respectively. The expected resulting difference in population size is 〈Δ〉 = (*β* − *δ*)*N*. We note that the linear dependency of *β* on *N* leads to *β* < 0 when *N* > *b*_0_ *K*/(*b*_0_ − *θ*). Values of *β* < 0 are interpreted here as death rates, such that δ* = |*β*| + *δ* for *β* < 0. In this way the convergence towards the logistic growth is fulfilled (see S2 Appendix).

The workflow during one time step *t*_*i*_ is given in Fig 2. All actions are simultaneous and independent. The synchronous update results in “overlapping generations”, i.e., individuals can survive several updates. The state of the system at time *t*_*i*_ depends only on the state at the previous time point *t*_*i*−1_. Thus, the model is a Markov process.

The time evolution of the probability *P*_*N*_(*t*) to have *N* individuals at time *t* is the Master equation:

$$\begin{array}{ccc} P_{N}\left(t+\tau \right)-P_{N}\left(t\right) & = & \sum_{\Delta =1}^{N}T\left(N|N-\Delta \right)P_{N-\Delta }+\sum_{\Delta =1}^{\infty }T\left(N|N+\Delta \right)P_{N+\Delta }-\\ & & \sum_{\Delta =1}^{N}T\left(N+\Delta |N\right)P_{N}\;-\;\sum_{\Delta =1}^{N}T\left(N-\Delta |N\right)P_{N}. \end{array}$$ (7)

The probability of transition from the state with *N* to the state with *M* individuals is denoted by *T*(*M*|*N*), and Δ ≡ |*N* − *M*|. For the classical one-step process only transitions including one individual Δ = 1 are allowed; instead, we allow all transitions 0 ≤ Δ ≤ *N*. (No more than *N* individuals can be born, as each individual can give birth at most to one newborn at each time step in our formulation.)

Multiple processes can lead to the same Δ for given *N* and *M*. For example, given two individuals *N* = 2, the transition from *N* = 2 to *M* = 3, Δ = 1, is possible by (i) birth of one new individual, or (ii) birth of two new individuals and death of one individual. The transition probability *T*(3|2) includes both (mutually exclusive) cases:

T(3|2)=(21)(20)β(1-β)δ0(1-δ)2+(22)(21)β2(1-β)0δ(1-δ). (8)

The first term on the right-hand side of Eq (8) describes the probability that case (i) happens. Here, *β* is the birth probability, (1 − *β*) is the probability that the second individual does not produce a new individual, (1 − *δ*)^2^ is the probability that none of the two individuals dies. The first (21) and the second (20) binomial coefficients reflect different combinations due to the birth and death process, respectively. The second term on the right-hand side describes the probability of case (ii) (the general formulation of transition probabilities is given in S2 Appendix).

This Master equation of our IBM approaches the deterministic logistic equation in the continuum limit *τ* → 0 and *K* → ∞ (see S2 Appendix).

### Growth-diffusion model

The growth-diffusion model tracks each individual as it decides on all three basic actions of birth, death, and movement. It follows that, in the appropriate limit *τ* → 0, Δ*x* → 0, *K* → ∞, the IBM of growth-diffusion approaches the Fisher-Kolmogorov equation. In a homogeneous environment, changing *K* can be seen as a coordinate rescaling since for a fixed population density in individuals/km^2^, a lower numbers of individuals in a cell corresponds to a higher spatial resolution (i.e. a smaller cell area). The frequency of updates, i.e., the inverse of the number of basic actions during one update, as well as the cell step size, i.e., Δ*x*, affect both movement and growth. Our IBM is formally similar to the discrete-time/discrete-space IBM studied in [12]. As in [12], individuals’ movements are modeled as random walks and the zero-noise limit of the growth term approaches the logistic model. However, the detailed stochastic model of growth differs from the model presented here (Eqs (5) and (6)) [12].

The two-dimensional FK equation is

$$\begin{array}{c} \frac{\partial \rho }{\partial t}=D\left(\frac{\partial^{2}\rho }{\partial x^{2}}+\frac{\partial^{2}\rho }{\partial y^{2}}\right)+r\rho \left(1-\frac{\rho }{K}\right) \end{array}$$ (9)

where *ρ* = *ρ*(*x*, *y*, *t*) is the density at position *x* and *y* and at time *t* [32]. Solutions give traveling waves of population expansion propagating asymptotically at constant speed. The minimal propagation speed is $v_{\text{det}}=2\sqrt{Dr}$, where the abbreviation “det” stands for “deterministic” [32].

The update protocol of our IBM, i.e., the growth term (Fig 2) “plus” the movement term (section), is equivalent to the Forward Euler integration in that updates are performed according to the system’s state at the beginning of each time step [33]. The limit *N* → ∞ of 〈*N*(*t* + *τ*)〉 − 〈*N*(*t*)〉 calculated with Eq (7) (see also Eq (28) in S2 Appendix) and Eq (22) in S1 Appendix is a Forward Euler integrator of logistic growth and diffusion, respectively. By modeling a real system, the spatial resolution Δ*x* and the temporal resolution *τ* should be chosen so that the resulting *D*, *b*_0_, and *d*_0_ can be correctly treated as probabilities. If this is true, the time step satisfies the Von Neumann criterion ([34]) which guarantees that the simulation is stable, i.e., numerical errors will not be amplified.

## Results of numerical simulations

Section studies the effects of discreteness, i.e., the fact that the population consists of discrete entities (individuals), with simulations of (i) the diffusion model, (ii) the growth model, and (iii) the growth-diffusion model. As mentioned in the introduction, such a discrete description of a population can capture stochastic effects [5].

### Diffusion term

We simulate the discrete-time one-dimensional random walk (see section, S1 Appendix), and compare the microscopic diffusion coefficients, i.e., the diffusion coefficient calculated using the microscopic parameters of a random walk such as *P*_move_, *P*_direction_, Δ*x*, and *τ* (see Eq (3)), with the macroscopic diffusion coefficient, i.e., the diffusion coefficient calculated using large-scale properties of the system such as the variance of positions of all individuals at a given time (see Eq (2)). Simulation parameters and results are given in Table 1. Since individuals move independently, the slope of the variance, which is a linear function of time, is an unbiased estimator of *D*. The estimated macroscopic diffusivities are within the range of the microscopic coefficients (see Table 1). The macroscopic and microscopic views are in agreement as expected.

**Table 1:** Simulation parameters used for the one-dimensional IBM of movement and resulting macroscopic diffusion coefficients. *N*_0_ = 3000 (i.e. simulations are far from continuous-time regime), Δ*x* = 1, and *τ* = 1. The corresponding microscopic diffusion coefficients are calculated using Eq (3). To estimate the macroscopic diffusion coefficients we measure the variance in the position of all individuals at different time points. Since the variance of the position is a time series we calculate its time derivative and estimate the mean of the derivative. The macroscopic diffusion coefficients are then estimated using Eq (2).

*P*_move_	microscopic *D*	mean of the derivative of Var(*x*)	macroscopic *D*
1	0.5	1 ± 0.1	0.5 ± 0.05
2/5	0.2	0.41 ± 0.05	0.205 ± 0.025
1/5	0.1	0.19 ± 0.02	0.095 ± 0.01

### Growth term

We focus on parameters *b*_0_, *θ*, and *K*. Hereafter, for ease of discussion, the death probability, *d*_0_ = 0.

#### Asymptotic behavior for increasing *K* and decreasing *θ*

**Fig 3:**
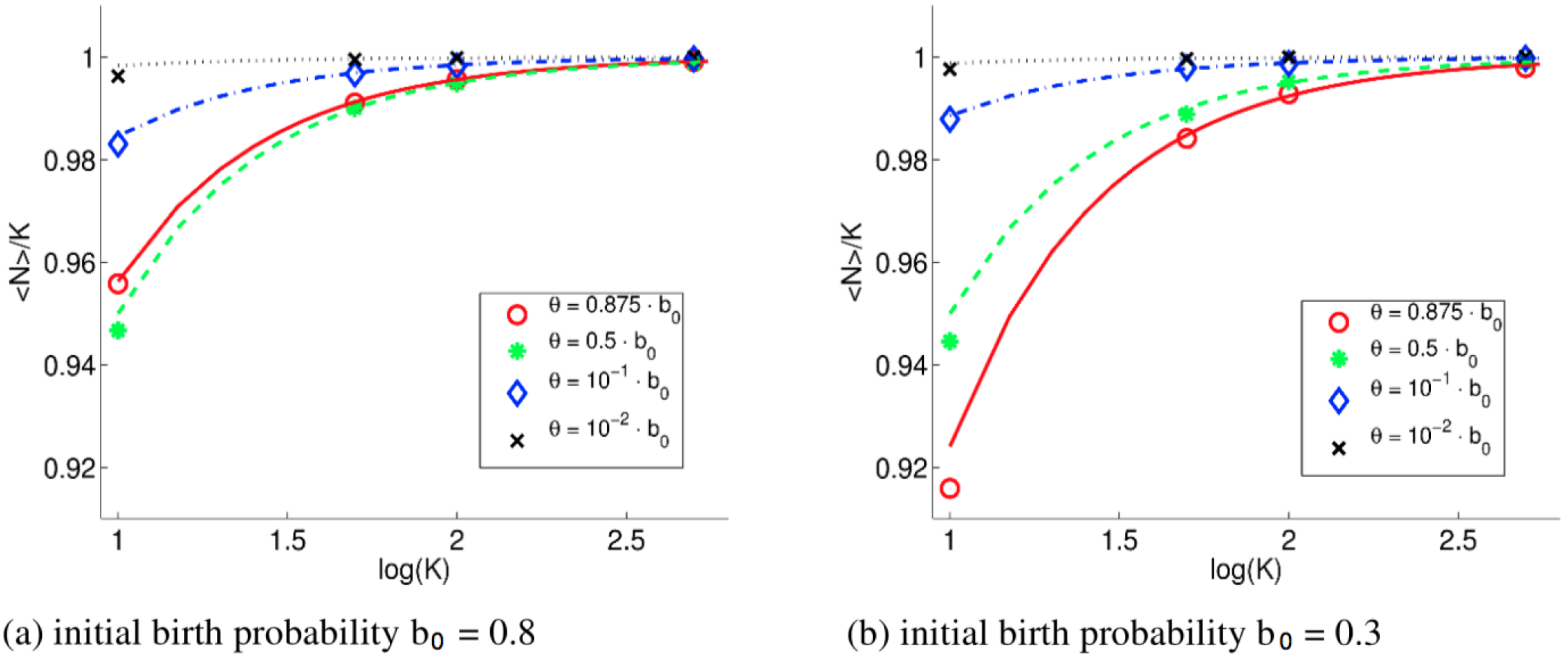
IBM of growth: The normalized effective carrying capacity 〈*N*〉/*K* depends on *K* and the turnover *θ*. Left panel (a) presents simulation results for *b*_0_ = 0.8 and *d*_0_ = 0. Symbols and colors indicate estimated 〈*N*〉/*K* for different turnover values shown in the legend. Lines represent the predicted effective carrying capacity 〈*N*〉 (Eq (11)) normalized by *K*. Right panel (b) presents simulation results for *b*_0_ = 0.3 and *d*_0_ = 0.

First, we investigate the influence of fluctuations around equilibrium for various choices of *K* and *θ*. Simulation parameters are given in Table 2. To estimate the effective carrying capacity 〈*N*〉, i.e., the number of individuals in the steady state, *N* is averaged over time in 100 realizations for each parameter set, Fig 3.

**Table 2:** Simulation parameters used for the IBM of growth. *d*_0_ = 0 for all parameter sets. The initial number of individuals for each simulation is *N*_0_ = *K*. The system is simulated for 500 time steps.

carrying capacity *K*	birth probability *b*_0_	turnover *θ*
10, 50, 100, 500	0.8	0.7
		0.4
		0.08
		0.008
	0.3	0.2625
		0.15
		0.03
		0.003

The resulting effective carrying capacity 〈*N*〉 is lower than *K*.

The lower 〈*N*〉 owes to the non-linear properties of the stochastic logistic equation:

$$\begin{array}{c} \frac{\text{d}N}{\text{d}t}=rN\left(1-\frac{N}{K}\right)+Z. \end{array}$$ (10)

*Z* is the component of (relative) fluctuations and 〈*Z*〉 = 0 [21]. Following Renshaw [21], and noting that, for our binomial process, *N* ∼ *K*, probability of birth or death ∼*θ*, we can estimate the effective carrying capacity as

$$\begin{array}{c} \left\langle N\right\rangle ∼K-\frac{2\theta (1-\theta )}{2r-r^{2}}. \end{array}$$ (11)

Fig 3 predicts that the relative deviation of 〈*N*〉 from *K* decreases with increasing *K*, as expected. Considering Eq (11): for increasing *K*, the deviation term becomes negligible, and 〈*N*〉/*K* → 1 as *K* → ∞.

If we decrease the turnover *θ* at constant *b*_0_, the relative deviation of 〈*N*〉 from *K* changes. We consider two examples: *b*_0_ = 0.3 and *b*_0_ = 0.8 (Fig 3). The direction of this change depends on both *θ* and *b*_0_. In the case *b*_0_ = 0.3, a decrease in *θ* corresponds to a decrease in the fluctuations, and 〈*N*〉/*K* → 1 monotonically. On the other hand, when *b*_0_ = 0.8, the behavior of the system is twofold: an initial decrease in *θ* from 0.7 to 0.4 leads to an increase of stochastic fluctuations, i.e., to a larger deviation of 〈*N*〉 from *K*. A further decrease in *θ* leads to a decrease fluctuations, and 〈*N*〉/*K* → 1.

To explain the dependence of the fluctuations on the turnover *θ* and on *b*_0_, the second term of Eq (11) can be rewritten for the ratio of *θ* and *b*_0_, *α* ≡ *θ*/*b*_0_:

$$\begin{array}{c} f=\frac{2\alpha (1-\alpha b_{0})}{2-b_{0}}. \end{array}$$ (12)

**Fig 4:**
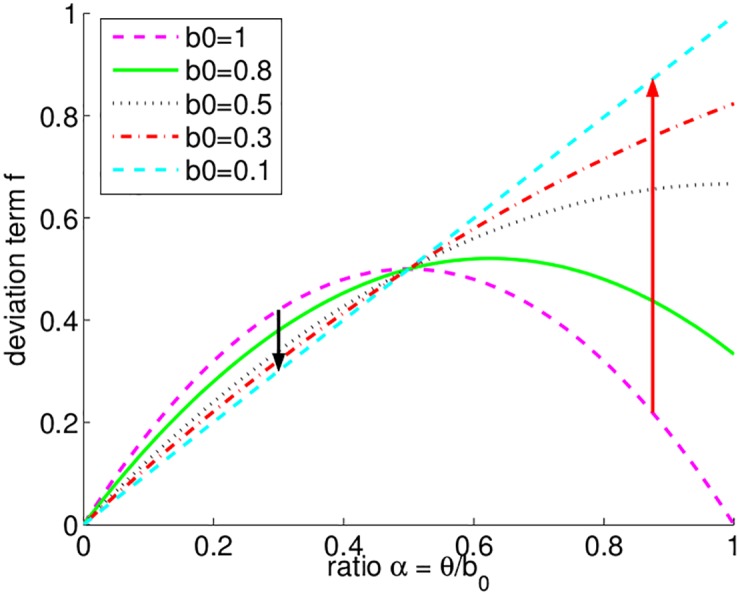
The term describing the deviation of 〈*N*〉 from *K* in dependency of the ratio *α* ≡ *θ*/*b*_0_ (Eq (12)). Different values of *b*_0_ are used (see legend for corresponding colors and line styles). As an example, the red arrow shows how the deviation *f* changes when *θ*/*b*_0_ = 0.875 and the *τ* is decreased. The black arrow shows how the deviation changes when *θ*/*b*_0_ = 0.3 and the *τ* is decreased.

Fig 4 illustrates the dependency of *f* on the ratio *α*. Here, *α* varies from 0 to 1, since we are only interested in the biologically-relevant case of competition-driven lowering of the per-capita birth rate with increasing population density (*θ* < *b*_0_).

We identify two regimes: *b*_0_ ≤ 0.5 and *b*_0_ > 0.5. When *b*_0_ is held constant and *b*_0_ ≤ 0.5, a decrease in the turnover *θ*, i.e., in the ratio *α*, always leads to a decrease in the deviation term *f* (Fig 4). When *b*_0_ > 0.5, the behavior of the system is more complex. With an initial decrease in the turnover, *f* increases until it reaches its maximum value. With a further decrease of the turnover, *f* decreases. We may intuitively explain this by realizing that the variance in a binomial process is maximal when its probability of success (in our case *θ*) is 0.5. Different choices of parameters (*b*_0_) position the system on either side of this maximum, and, as a consequence, can give rise to opposite behaviors of the fluctuations for decreasing *θ*.

#### Convergence towards the one-step process

Here we indicate that by “slowing down” the IBM, i.e., decreasing *τ* and lowering the expected number of events within one update, the discrete-time model approaches the continuous-time birth-death process. To accomplish this, we change *b*_0_ and *θ* while keeping their ratio constant. Note that this does not correspond to studying the same model at different time resolutions; rather, we are comparing IBMs with different update frequencies but same continuum limit (up to a rescaling of *τ*).

**Fig 5:**
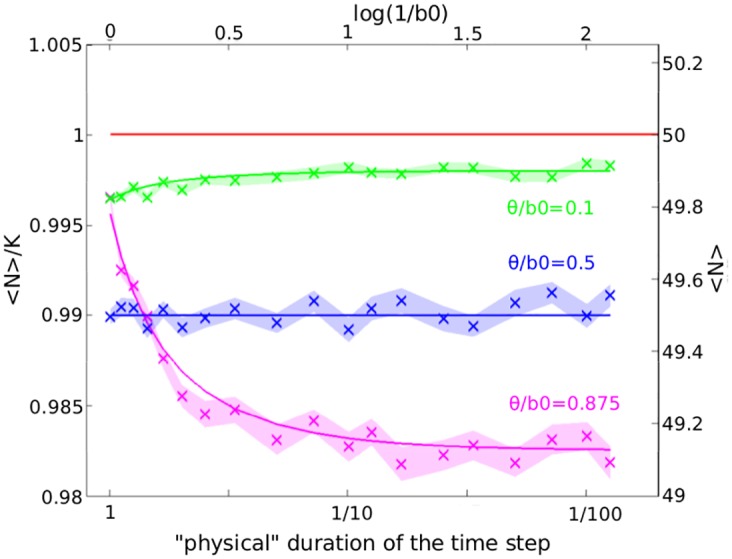
IBM of growth: The effective carrying capacity 〈*N*〉 depends on the frequency of system updates. The time step *τ* is changed by changing *b*_0_ and *θ* keeping their ratio constant (*d*_0_ = 0). A decreasing “physical” duration of *τ* corresponds to increasing values of log(1/*b*_0_). The carrying capacity is *K* = 50. 50 realizations are performed for each set of parameters as indicated in the image. Shaded areas correspond to 〈*N*〉 ± *SEM* estimated in the simulations where *SEM* is the standard error of the mean. Solid lines correspond to the predicted 〈*N*〉 (Eq (11)). The red solid line corresponds to the deterministic case 〈*N*〉 = *K*.

We explore the parameter space by spanning two orders of magnitude in *θ* and *b*_0_. Three different ratios of *θ*/*b*_0_ are considered; *θ*/*b*_0_ = 0.875, 0.5, and 0.1. The total number of time steps is inversely proportional to *b*_0_ and *θ* to fix the total simulation time. Further simulation details and simulation results are given in Fig 5, as well as the comparison with the approximation Eq (11) (three solid lines).

When *θ*/*b*_0_ = 0.5, the effective carrying capacity 〈*N*〉 does not change as a function of the duration of the time step. Approximation Eq (11) predicts that, if *θ* = 0.5 ⋅ *b*_0_, the deviation of 〈*N*〉 from *K* is constant: 〈*N*〉 = *K* − 1/2. In Fig 4, this case corresponds to the intersection of all curves for different values of *b*_0_ at *θ*/*b*_0_ = 0.5.

When the time step is small and *θ* → 0, the term describing the fluctuations *Z* is a Poisson distributed variable with Var(*Z*) = 2 *Kθ* that is monotonic in *θ*. Eq (11) then becomes 〈*N*〉∼*K* − 2*θ*/(2*r* − *r*^2^) which corresponds to the approximation of 〈*N*〉 in the continuous-time one-step process [21] and agrees with simulation results (Fig 5). In this regime, 〈*N*〉 becomes independent of *τ*.

Thus, the properties of the growth IBM around the equilibrium can be can be summarized using Eq (11). By normalizing Eq (11), we get

$$\begin{array}{c} \frac{\left\langle N\right\rangle }{K}=1-\frac{1}{K}\frac{2\theta (1-\theta )}{2r-r^{2}}\;. \end{array}$$ (13)

**Fig 6:**
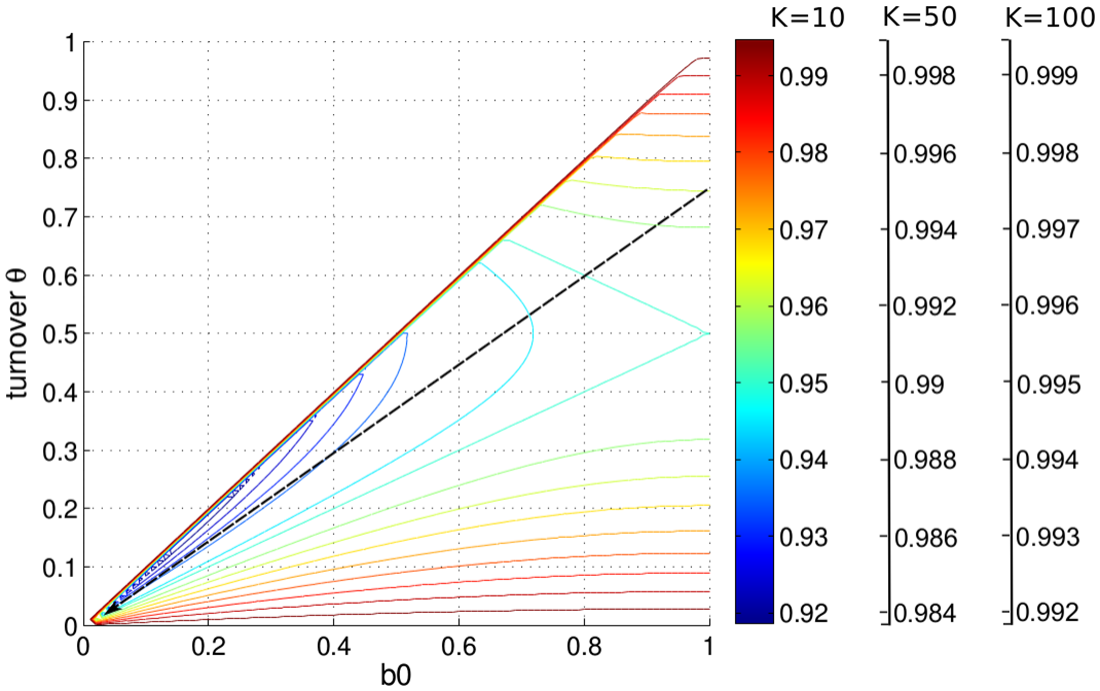
Contour plot of 〈*N*〉/*K* in dependency on *b*_0_ and the turnover *θ*. The contours (colored solid lines) are calculated using Eq (13). The pattern of the dependency of 〈*N*〉/*K* on *b*_0_ and *θ* remains the same for different *K*, but 〈*N*〉/*K* → 1 when *K* → ∞. Here, color legends are given for *K* = 10, *K* = 50, *K* = 100. The dashed black arrow shows how 〈*N*〉/*K* changes when the time step decreases and *θ*/*b*_0_ = 0.6/0.8. The arrow head points towards the region of smaller time steps. The region *θ* > *b*_0_ is not allowed.

The normalized effective carrying capacity 〈*N*〉/*K* is shown as a two-dimensional contour plot in Fig 6. By considering the behavior of IBMs with more and more frequent updates, the trajectory of 〈*N*〉/*K* (Fig 6) can be represented by straight lines going towards the origin with slope determined by *θ*/*b*_0_. For example when *θ*/*b*_0_ = 0.6/0.8, a decrease of the time step leads to an increase of the noise, i.e., 〈*N*〉/*K* declines (trajectory shown by a dashed black arrow).

### Growth-diffusion model

#### One-dimensional case

The propagation speed *v* of the emerging traveling wave is a fundamental macroscopic property of the FK equation. We thus investigate the relation between *v* and the carrying capacity *K*, which is the parameter regulating fluctuations in continuous-time reaction-diffusion systems [22–24, 26]. In our simulations, *K* varies from 10 to 1000 individuals and *b*_0_ = 0.8, 0.3, 0.1, and 0.01; *θ* = 10^−1^ ⋅ *b*_0_; *d*_0_ = 0. The diffusion coefficient—as determined by microscopic parameters of the random walk (see section)—is set to *D* = 0.2. The initial number of individuals is *N*_0_ = *K*, and their initial position is *x*_0_ = 0. The simulation is stopped when the individuals reach *x* ≈ 300. Ten repetitions are performed for each parameter set.

These parameters are relevant for ecological dynamics, especially with respect to hunter-gatherer populations. For example, *K* in the range of 10 to 1000 individuals is consistent with hunter-gatherer group sizes [35]; the mean size of modern hunter-gatherer groups is ∼ 50 individuals, with a maximum of ∼ 270 and minimum of ∼ 13 individuals [36, 37]. The population density of modern hunter-gathers is ∼0.01 − 9.5 individuals/km^2^ [37], so the spatial resolution of our simulations corresponds to 1 km^2^-100 km^2^. Growth rates in the present simulations range between 0.01 and 0.8, whereas the intrinsic growth rate of foragers is ∼0.02/year [37].

We estimate the speed *v* by tracking the wave front position defined as *x*_*K*/2_ where *N* = *K*/2. We measure *v* after the system stabilizes to linear speed (see section).

**Fig 7:**
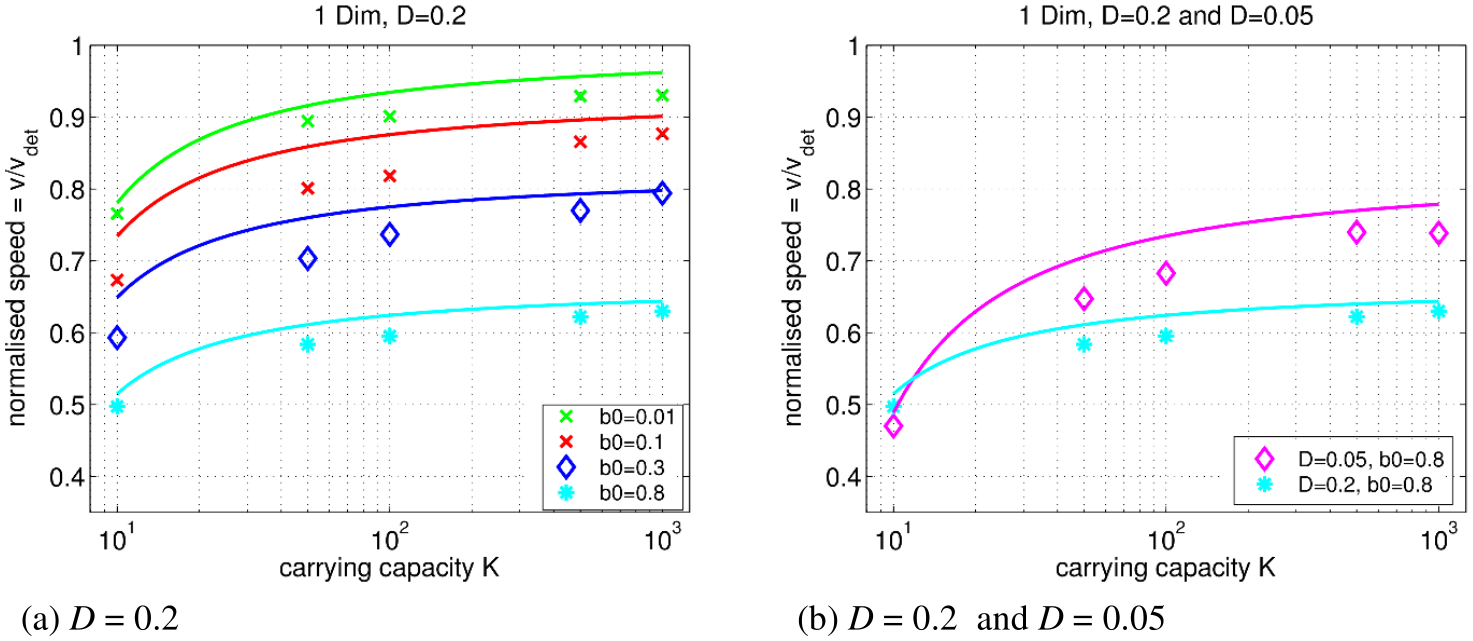
IBM of growth-diffusion: The relation between the normalized speed *v*_norm_ and *K* in the one-dimensional case. Left panel (a) presents *v*_norm_ when *D* = 0.2. Symbols show the measured normalized speed (see legend; *θ* = 10^−1^ ⋅ *b*_0_; *d*_0_ = 0). Solid lines show the analytical approximation Eq (15) in respective colors. Right panel (b) presents the effect of *D*. Lines show the analytical approximation (Eq (15)) for *D* = 0.2 and *D* = 0.05 respectively. *b*_0_ = 0.8 in both cases. Typical error bars are less than 1%.

Fig 7 shows the relation between *v*_norm_ = *v*/*v*_det_ (see) and *K*. For all our parameter sets, the estimated speed *v* is lower than *v*_det_ and depends on *K*, contrary to the deterministic FK model, but seen previously in stochastic models [22–24, 26].

The lower propagation speed owes to the behavior at the tip of the traveling wave [22–24, 26]. A stochastic population with a lower number of individuals is more likely to go extinct due to fluctuations. At the tip of the traveling wave, *N* ≪ *K*, so extinctions of new “colonies” slow the wave front propagation [26].

To summarize, our parameter space exploration reveals tree types of asymptotic behavior toward higher normalized speed *v*_norm_. Increasing *K* decreases relative fluctuations leading to fewer extinction events. At *K* ≥ 500, *v* reaches a plateau at values smaller than *v*_det_: *K* → ∞ is not sufficient to reach the continuum limit.

Next, the normalized speed increases when growth and movement probabilities are decreased. The dependency of *v*_norm_ on *r* and *D* can be explained by the leading edge approximation of the traveling wave solution [28, 38]. Although the microscopic update rules of stochastic population-based models and our discrete-time IBM are different, they both have multiple births, deaths, and movements in a single time step.

The dispersion relation derived in [38] can be readily applied to our model, and it is quoted here for convenience:

$$\begin{array}{c} v\left(\gamma \right)=\frac{1}{\gamma \cdot \tau }\ln\left[1+D^{\prime }\left(e^{-\gamma \Delta x}-1\right)+D^{\prime }\left(e^{\gamma \Delta x}-1\right)+\left(b_{0}-d_{0}\right)\right], \end{array}$$ (14)

*D*′ = *P*_move_(1/*n*_neighbors_) is the diffusion probability (Eq (3)). The front of the FK wave travels at the minimal speed where d*v*/d*t* = 0 (know as the “pulled” wave type) [28, 32]. This minimal speed approaches *v*_det_ when decreasing the growth probability(see Fig 7(a)) or decreasing *D*′ (see Fig 7(b)).

The approximation *v* ∼ min *v*(*γ*) (Eq (14)) is valid only in the limit *K* → ∞. However, [28] find an approximation that includes the impact of the carrying capacity *K*. We quote this result here as well:

$$\begin{array}{c} v=v\left(\gamma_{0}\right)-\frac{\pi^{2}v^{\prime \prime }\left(\gamma_{0}\right)}{2L^{2}} \end{array}$$ (15)

[28]. *γ*_0_ is the parameter that minimizes the dispersion relation for *v*. *v*′′(*γ*_0_) is the second derivative of *v* with respect to *γ* at *γ*_0_. The correction term *L* includes the dependency on ln *K*.

We compare approximation Eq (15) with *v*_norm_ estimated in our model. The approximation is given by solid lines in Fig 7. The simulated *v*_norm_, especially in the limit of increasing *K*, is in rough agreement with Eq (15) as we expected, given the strong fluctuations regime we are considering, appropriate for ecological systems. Furthermore, approaching the limit of continuous time of the growth term (*b*_0_ < 0.1) and with an increased carrying capacity (*K* ≥ 500), the estimated speed approaches asymptotically the deterministic speed. This asymptotic convergence to *v*_det_ is consistent with previous results on continuous-time FK equation [22–24, 26].

Note that the *K* → ∞ limit of the reaction-diffusion IBM agrees with a slow-down of the wave due to the Forward Euler time step. In particular, the slow-down is pronounced at high *r* and *D* (see also Fig 7 and the approximation Eq (14)).

#### Two-dimensional case

We perform simulations on a 1000x100 rectangular grid. The values of *K*, *b*_0_, *d*_0_, *θ* are equal to the parameters used for the one-dimensional case, and *D* = 0.2. The individuals are confined to a 1000 by 100 cells domain. At the beginning of the simulation (*t* = 0), individuals are arranged in a line parallel to the y-axis, at *x*_0_ = 0. We use this “line setup” due to its comparability with the one-dimensional case with a traveling wave moving in one direction. The initial number of individuals is *N*_0_ = *K* in each of these grid cells. In accordance to the one-dimensional case, the simulation is stopped when the individuals reach *x* ≈ 300.

We calculate the *y*-averaged wave profile along the *x*-axis, i.e., the mean number of individuals at a given position *x*, and, in analogy to the one-dimensional case, the speed is determined by estimating *x*_*K*/2_ as a function of time.

**Fig 8:**
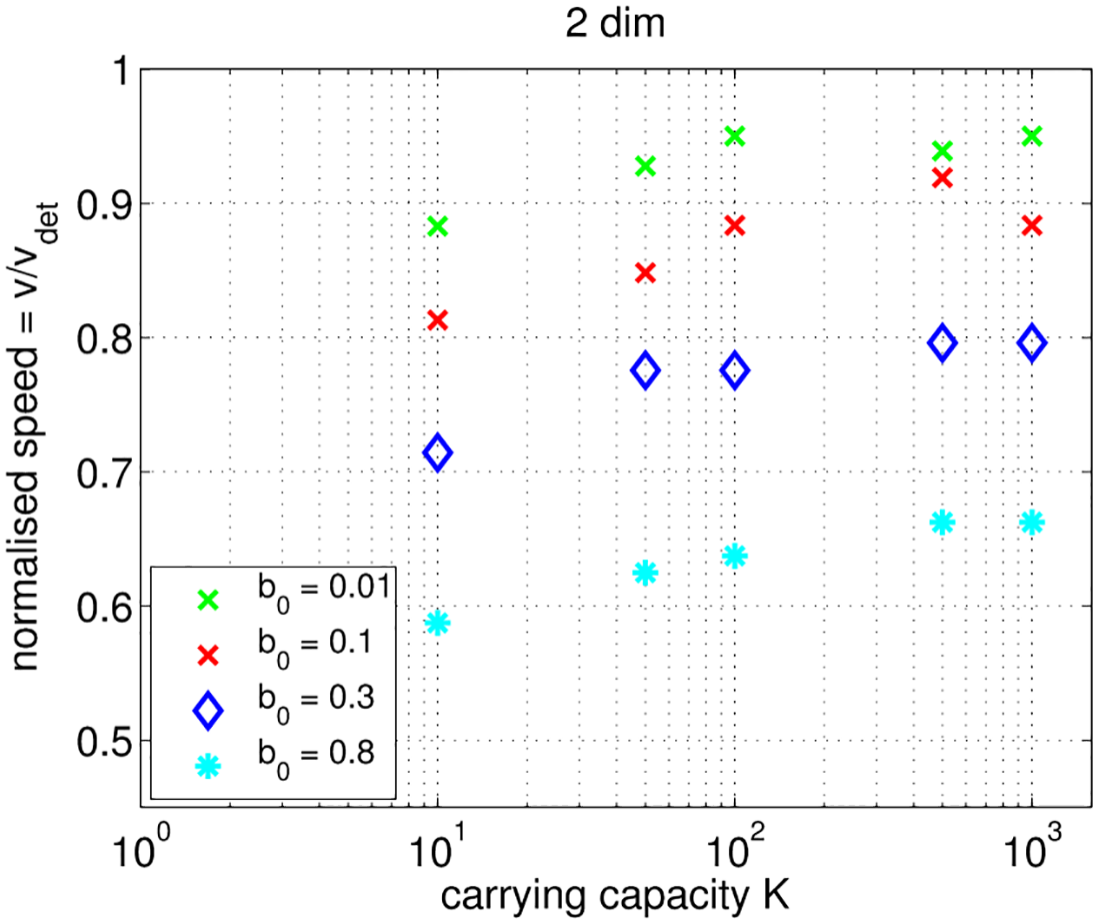
IBM of growth-diffusion: The relation between the normalized speed *v*_norm_ and the carrying capacity in the two-dimensional case. Colors and symbols indicate *b*_0_ values shown in the legend. *D* = 0.2, *θ* = 10^−1^ ⋅ *b*_0_, and *d*_0_ = 0.

Results are presented in Fig 8.

As in the one-dimensional case, the speed *v* is consistently lower than the deterministic speed *v*_det_. Furthermore, *v* increases with (i) an increase of the carrying capacity *K* and (ii) a decrease of *r*: the two asymptotic behaviors discussed for the one-dimensional case can still be identified. Additionally, the speed is higher in the two-dimensional case compared with the one-dimensional case. The difference is especially pronounced for *K* ≤ 50 and *b*_0_ ≥ 0.3 corresponding to stronger fluctuations in the number of individuals. In the one-dimensional case, the wave is momentarily halted when the population in the farthest nonempty cell at the (zero-dimensional) tip of the wave goes extinct. In the two-dimensional case, the propagation of the wave is slowed down, but is not stopped completely since the probability that extinction happens in all cells at the (one-dimensional) front of the wave along the y-axis is very low. Furthermore, in the two-dimensional case, “extinct” cells at the wave front can be populated by non-extinct neighbors on each side. This effect is enhanced by the higher total number of individuals in the system. Thus, the extinction at the tip of the wave has a more dramatic effect on the one-dimensional domain.

### Dispersal into the Americas

Understanding the timing and the dynamics of the colonization of the Americas by modern humans during the late Pleistocene is a challenging problem. Recent genomic evidence shows that all Native Americans diverged from their Siberian ancestor 20 ka (1 ka—“kilo-annum”– represents 1000 years before present) and no earlier than 23 ka [39]. The time of population differentiation supports the hypothesis that people could reach southern South America by 14.6 ka (site Monte Verde), before the Clovis sites in North America were established (around 13 ka) [39–41] (see also Table 3). Archeological sites like Schaefer, Hebior, and Page-Ladson also suggest a human presence in North America by 14.6 ka [42, 43]. Other important archeological sites with radiocarbon dates are given in Table 3. In the context of the timing of the colonization, the opening of ice-free corridors in the Laurentide and Cordilleran ice sheets that covered North America played a crucial role. Namely, the coastal corridor opened around 16 ka and the interior corridor around 14 ka [39].

**Table 3:** Archaeological dates and simulated arrival times during the late Pleistocene dispersal in the Americas. Dates are given in thousand years before present (ka). The second column shows archaeological dates and the corresponding references. The subsequent columns show the difference between the simulated arrival times and the archaeological dates for the used sets of parameters. Here, the earliest archaeological date is used to calculate the difference in case that a range of dates is presented for a site. Positive values of the difference indicate that the individuals arrive at the site earlier than shown by the archaeological data. Negative values indicate that the individuals arrive latter.

Site name	achaeol. date	set 1	set 2	set 3	set 4
Schaefer, Hebior sites (Wisconsin)	14.8–14.2 [42]	0.6	0.7	0.6	0.4
Paisley Cave (Oregon)	14.5–14 [44]	1.1	1.1	1	0.9
Meadowcroft Shelter (Pennsylvania)	14.5–14 [44]	-0.2	-0.2	-0.4	-0.5
Buttermilk Creek, Friedkin site (Texas)	15.5–13.2 [44]	-0.2	-0.1	-0.3	-0.4
Page-Ladson site (Florida)	14.6 [43]	0.6	0.6	0.4	0.3
Piedra Museo (Argentina)	13.1– 12.9 [42]	0.5	0.4	0	-0.2
Quebrada Santa Julia (Chile)	13.1 [42]	0.6	0.6	0.2	0
Monte Verde (Chile)	14.6–14.2 [44]	-0.9	-1	-1.4	-1.6
Fell’s Cave(Chile)	13.1– 12.9 [42]	0.4	0.3	-0.1	-0.3
Mean squared error		3.59	3.72	3.78	4.16

In the following, we explore the properties of the IBM model with a suite of simulations of the colonization of the Americas. The model corresponds to a basic scenario involving as few parameters as possible. First, we test the match between the simulated arrival times and empirical data on the age of archaeological sites. Further, we check if the stochastic fluctuations contribute significantly to the simulation results for the relevant range of parameters. In other words, we investigate if and how the resolution of the stochastic IBM simulation changes the propagation speed and thus the arrival times.

Simulations are performed on a global spherical grid based on a tessellation of a regular icosahedron. Most of the grid’s nodes have 6 neighbors, i.e., spatial elementary units are hexagons. Exceptions on the entire spherical grid are 12 nodes that have only 5 neighbors. (The altitude maps are produced using a global relief model [45].)

As previously mentioned, individuals live on the grid’s cells. Furthermore, on cells, environmental variables such as altitude and ice cover are defined. We use paleo-topography data [46] and update ice cover and see level every 500 years. The movement of individuals is restricted now to the “habitable” cells: individuals do not move into the sea, to cells whose altitude is higher than 2500 m, and to cells covered by ice. In this implementation of the model, the movement probability is a function of both accessible and inaccessible cells, according to the following equation

$$\begin{array}{c} P_{\text{move}}^{\text{new}}=P_{\text{move}}-\frac{N_{\text{inaccess}}}{N_{\text{tot}}}\;, \end{array}$$

where $N_{\text{tot}}$ is the total number of the neighbors and $N_{\text{inaccess}}$ is the number of inaccessible neighbors. This ensures that the local diffusion coefficient, in the direction where movement is possible, is not affected by the anisotropy of the surrounding environment.

We use four different sets of parameters (see Table 4). The last column of Table 4 shows the total number of individuals at the end of each simulation.

**Table 4:** Parameter sets used for the simulations of the late Pleistocene dispersal in the Americas. *K* is the carrying capacity of every cell. *N*_tot_ is the total number of individuals in the Americas at the end of the corresponding simulation. For all parameter sets, the resulting growth rate is 0.06/yr, the diffusion coefficient is *D* ∼ 180 km^2^/yr, and the deterministic speed is *v*_det_ ∼ 6.57km/yr. Δ*x* is the distance between two neighboring nodes, i.e., the spatial resolution.

parameter set	*τ*	Δ*x*	*K*	*ρ*	*θ*	*P*_move_	*N*_tot_
1	1 yr	60 km	307	0.1	0.03	0.2	3.7 ⋅ 10^6^
2	1 yr	30 km	77	0.1	0.03	0.8	3.7 ⋅ 10^6^
3	5 yr	60 km	307	0.1	0.15	1	3.7 ⋅ 10^6^
4	1 yr	60 km	10	0.003	0.03	0.2	1.1 ⋅ 10^5^

In the reference simulation (set 1), the spatial resolution is Δ*x* ∼ 60 km, leading to a cell area of ∼3700 km^2^. The temporal resolution is *τ* = 1yr.

The diffusion coefficient, the growth rate, and the carrying capacity (and the resulting speed) are chosen to be consistent with the estimated large-scale parameters of hunter-gatherer groups (see also section) [35–37]. For instance, the growth rate at the half of the carrying capacity is set to 3% per year, (which we assume is also equal to the turnover, giving an average individual lifespan of 33 yr when *N* = *K*), what corresponds to the growth-rate of a non-expanding population [35]. The diffusion coefficient is *D* ∼ 180 km^2^/yr. The resulting deterministic speed is *v*_det_ ∼ 6.57 km/yr. This value is in the middle of the estimated colonization speeds in the Americas during the late Pleistocene, namely 5-8 km/yr [47]. This propagation speed in the Americas is very high compared to other regions, e.g., 0.5 km/yr is the estimated speed of the colonization of Europe by anatomically modern humans [47]. Finally, the carrying capacity is chosen so that the resulting density is around 0.1 individuals per km^2^.

We run a second IBM with different spatial resolution: Δ*x* ∼ 30 km (parameter set 2, Table 4). In a third simulation, we change the time resolution, whereby one simulation step corresponds to 5 years (parameter set 3). Note, that the large-scale parameters *r*, *θ*/*τ*, *D*, *v*, and *ρ* are the same for all three IBMs.

Finally, we consider a setting with a low carrying capacity *K* = 10, leading to a population density of *ρ* = 0.003 individuals / km^2^ (parameter set 4). Compared to sets 1-3, the population density changes. Other large-scale parameters stay the same.

The dispersal into the Americas starts 18 ka from one occupied grid cell located in Beringia (latitude: 64° N, longitude: 159° W). The initial number of individuals in this cell is *N*_0_ = *K* for each set of parameters (Table 4). Thus, the colonization starts from only one group of individuals and there is no prolonged invasion from Beringia that would replace prior populations.

Model simulations are performed with the QHG code [31]. Due to the discrete-time algorithm, simulations can be parallelized, e.g., the QHG code uses shared-memory parallelization [31]. By using 32 threads, the reference simulation runs ∼12 times faster (∼45 min), than on a single thread.

**Fig 9:**
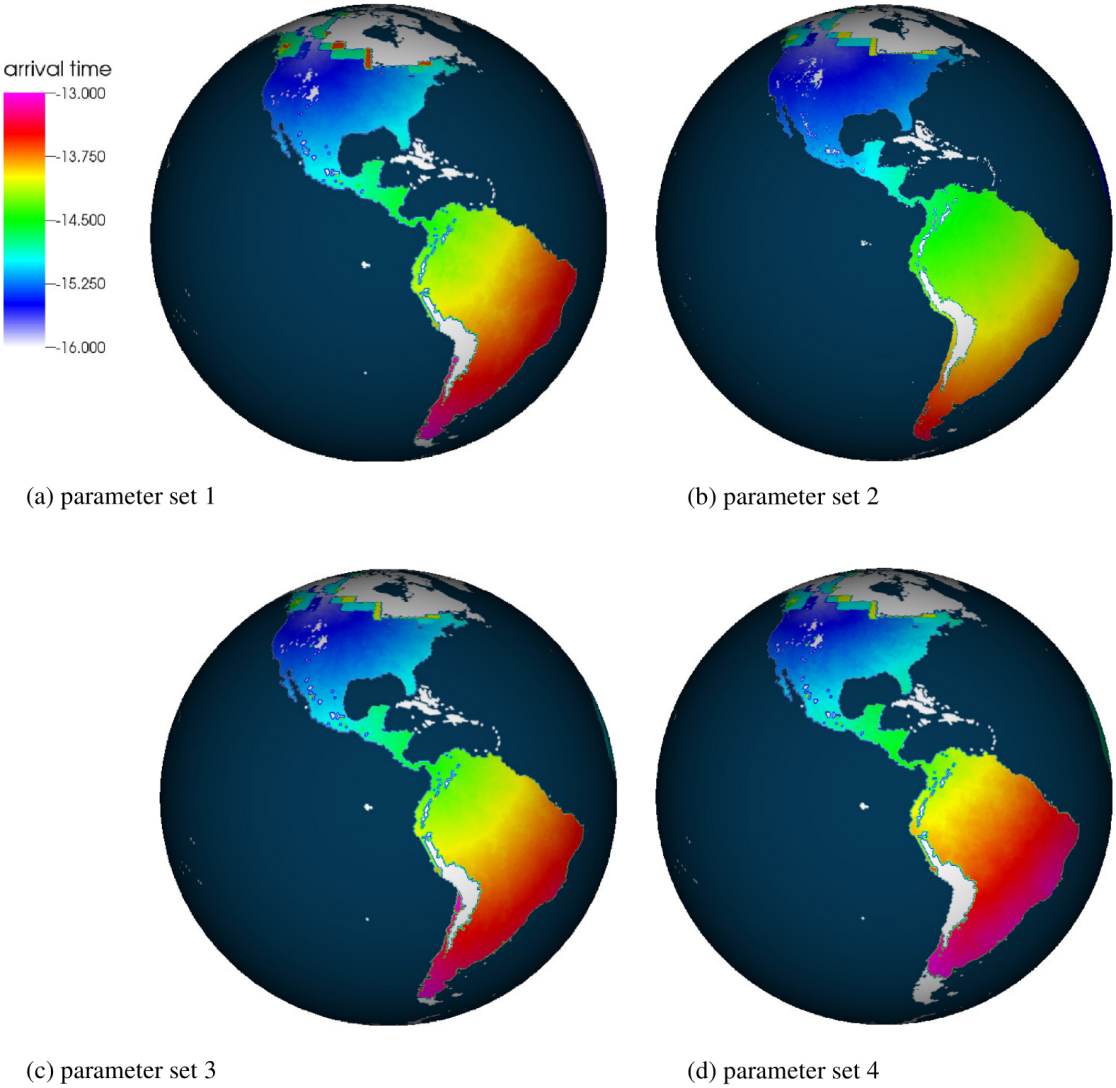
Maps of the arrival times. The snapshots are taken at 13 ka (1 ka—“kilo-annum” represents 1000 years before present). Thus, the latest arrival times correspond to 13 ka (shown in magenta). The earliest arrival times correspond to 16 ka (in light blue). Panels (a), (b), (c), and (d) show simulations with the parameter sets 1, 2, 3, and 4, respectively. Color codings of the arrival times are given in the legend. The white area corresponds to the land area without individuals: ice covered territory, mountains, and the territory that is not yet occupied, e.g., the white area in Patagonia (especially pronounced on panel (d) due to a slower wave).

**Fig 10:**
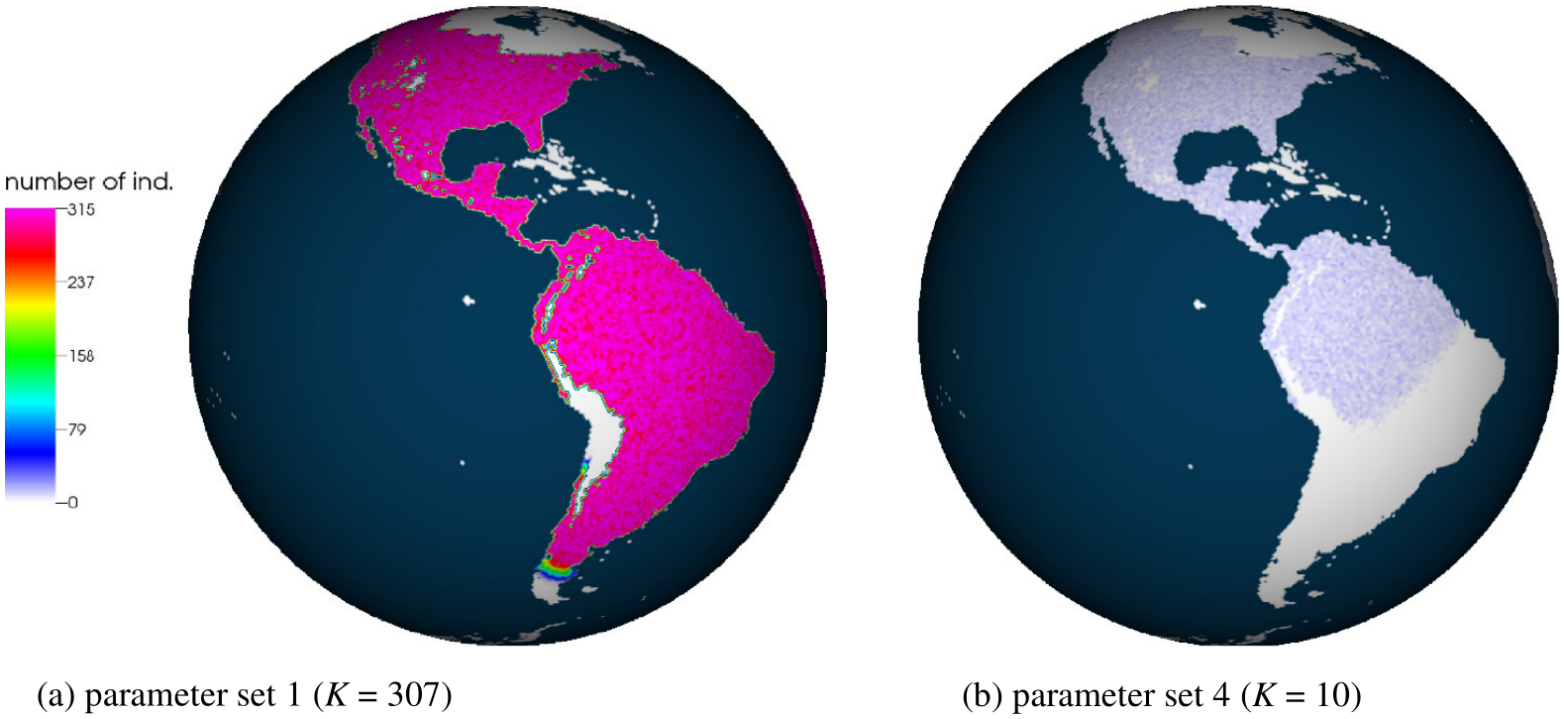
Snapshots of the simulations at *t* = 13.5 ka (2.5 ka after the ice-free corridor opens). The left panel shows the simulation with the parameter set 1, the right panel shows the simulation with the parameter set 4. The number of individuals *N* per cell is shown. Color coding for *N* is given in the legend. Furthermore, the sea is colored dark blue and the unoccupied territory is colored white. Panel (b) indicates that the wave does not have enough time to reach Patagonia, i.e., a large territory in South America remains unoccupied (white coloring of the territory).

During the simulations, the arrival time is recorded at each cell, i.e., the time at which the cell is colonized for the first time. Archeological dates and simulated arrival times at selected sites are given in Table 3. Fig 9 shows maps of overall arrival times at the time when individuals reach the southern tip of South America (*t* = 13 ka). Fig 10 predicts how far individuals disperse at *t* = 13.5 ka, i.e., 2.5 ka after the ice-free corridor opens. (In all our simulations, the corridor opens at 16 ka.)

The simulation with parameter set 1 predicts that individuals need ∼3 ka after the opening of the ice-free corridor to reach Patagonia (Figs 9 and 10, Table 3). In general, the arrival times at particular sites are in the range of archaeological estimates, or individuals arrive earlier (e.g. Paisley Cave, see Table 3). An exception is the Monte Verde site at which individuals arrive ∼1000 years later than predicted. As discussed in the literature, it is difficult to construct a FK-inspired model so that the population reaches Monte Verde on time or earlier [47]. This even holds true when the deterministic propagation speed is very high, e.g., 8 km/yr [47]. To fulfill the archaeological arrival times at Monte Verde with the IBM, one might need to increase the diffusion coefficient or to change the assumptions of the movement term, e.g., by considering coastal migration with Lévy-flight properties [48, 49].

Similar results and timings are obtained when the spatial resolution increases (parameter set 2).

When the time resolution is decreased, e.g., 1 simulation step corresponds to 5 years, the propagation speed slows down due to increased stochastic fluctuations (parameter set 3, see also section). Southern south America is reached ∼400 years later than in the case of a higher time resolution (parameter set 1). Thus, this low time resolution introduces an additional effect to take into consideration when running an IBM. Even a stronger slowdown of the wave can be observed when the carrying capacity is reduced to *K* = 10 (parameter set 4). In this case, Patagonia is reached ∼700 years later than in the simulation with parameter set 1. Thus, the relative amount of stochastic fluctuations plays a significant role in the simulations’ outcome. Assuming a starting date of 18 ka for the dispersal into the Americas, this corresponds to ∼4% of the total time required to colonize these continents.

## Discussion and conclusions

We introduced a discrete-time IBM for the diffusion, the logistic growth, and the logistic growth-diffusion processes. The IBM of movement is a discrete-time synchronous random walk which yields the diffusion equation in the continuum limit.

The IBM of growth uses a constant time step where per-capita birth and death probabilities are linear functions of the local density of individuals. Multiple births and deaths are possible at each time step. This stochastic model approaches the logistic equation in the continuum limit.

Analyzing the growth term, we found an analytical approximation of the effective carrying capacity 〈*N*〉

$$\begin{array}{c} \left\langle N\right\rangle ∼K-\frac{2\theta (1-\theta )}{2r-r^{2}} \end{array}$$ (16)

that agrees with simulation results. We verify that the effective carrying capacity is lower than *K* (Eq (16)). Furthermore, because the fluctuation term 2*θ*(1 − *θ*)/(2*r* − *r*^2^) is independent on *K*, an increasing *K* reduces relative fluctuations and 〈*N*〉/*K* → 1 (see also Eq (13)). These features are well-known for stochastic population-based models of growth [21, 50].

To summarize new results, we found that when the time step decreases, the dynamics of the noise term depends on the ratio *θ*/*b*_0_ owing to the binomial nature of the model. Furthermore, when the time step decreases, our discrete-time IBM approaches the Poisson distribution characteristic of the continuous-time birth-death process.

In summary, when *K* > 50, the deviation of the effective carrying capacity 〈*N*〉 from *K* is less than 2%. When *K* < 50 the deviation is more pronounced; e.g., when *K* = 10, 〈*N*〉 differs from *K* by 9%.

By combining the growth and the movement term, we get a discrete-time IBM of the growth-diffusion process that is the FK equation in the continuum limit. We reproduce the well-known phenomenon that the propagation speed of the traveling wave is lower than the minimal deterministic speed *v*_det_ and depends on the number of individuals in the system [22–24, 26, 28, 38]. In particular, the normalized speed *v*_norm_ = *v*/*v*_det_ depends on the carrying capacity *K*, the growth rate *r*, and the diffusion coefficient *D*. Simulation results match qualitative trends found for stochastic birth-death models, e.g., 〈*N*〉 < *K*, and stochastic growth-diffusion models, e.g., the dependency of *v* on *K*. However, the discrete-time algorithm indicates quantitatively different properties compared to continuous-time stochastic models (Figs 7 and 8).

Due to the discrete-time algorithm, simulations can be parallelized, which allows the consideration of the large-scale dynamics and high numbers of individuals. We apply the IBM to study the late Pleistocene expansion of the modern humans in the Americas, using millions of simulated individuals (Table 4). Even though only constant parameters *D*, *r*, and *K* are used, the IBM can reproduce most of the estimated arrival times. However, as for the idealised 1 and 2 dimensional cases, stochastic fluctuations affect the simulations’ outcome. For example, we indicate that when the time resolution decreases, i.e., stochastic fluctuations increase for the given parameters, the propagation of the individuals slows down and Patagonia is reached ∼400 years later than in the case of a higher time resolution. When an extremely low carrying capacity, *K* = 10, is used, the fluctuations increase as well. In this case, Patagonia is reached ∼700 years later than in the reference simulation.

The question is now whether such effects of stochasticity are a desirable property of a model of growth and dispersal, and to what extent. The discrete-time and the continuous-time representations are two regimes of population models: ecological data from systems under consideration are needed to evaluate the appropriate regime and to compare fluctuations in a real population with the predicted noise, depending on the spatial and temporal scales of the biological system. Moreover, when investigating concrete ecological questions, for example the dispersal of a certain species in an unoccupied territory [31], the effect of discreteness is an important feature. An important refinement of the IBM would be that individuals do not disperse homogeneously in space but live in groups of high density, e.g, herds, flocks, families, etc [51].

All parameters of our model can be made space and time dependent. Thus, this IBM can include an inhomogeneous environment (*K* ≠ const) and anisotropic diffusion, as well as evolutionary processes.

It is also possible to incorporate genetic information in the IBM and evaluate the evolution of the genetic structure of the population. Since our model follows individuals explicitly on large spatio-temporal scale, it is particularly suited to this type of genetic study. This approach can complement the more standard stepping-stone framework. Stepping-stone models are widely used for simulations to understand genetic consequences of range expansions; they are also based on reaction-diffusion systems [52] but do not follow individuals, each update step represents a non-overlapping generation, and movement and logistic growth are typically not stochastic, e.g., there are no oscillations around carrying capacity [44, 52–54].

The present IBM can also be used to evaluate emergent large-scale properties of a more elaborate agent-based framework, e.g., an IBM that considers survival and reproduction probabilities depending on the age and sex of individuals, thus bridging the gap between large-scale classical model and biologically accurate bottom-up models.

With synchronous updating of independent individuals, the model performs well on High Performance Computers (HPCs), contrary to implicit schemes. Thus, our model allows scaling to high spatial and temporal resolution, large domains, long simulation times, and large populations as we indicated in the application to the American dispersal of *Homo sapiens*. The model can be used for the simulation of large populations expansions on evolutionary timescales, and would allow for the inclusion of genetic information and the extension to more realistic biological models. This, in turn, would lead to significant progress in the understanding of the dynamics and genetic consequences of individual-based stochastic population dynamics.

## Supporting information

S1 Appendix Limit behavior of the diffusion term. (PDF)

S2 Appendix Limit behavior of the growth term. (PDF)
