# Supplementary material for: Individual-based modelling of population growth and diffusion in discrete time
Source: PLoS One. 2017 Apr 20;12(4):e0176101. doi: 10.1371/journal.pone.0176101 (PMC5398609; doi:10.1371/journal.pone.0176101)
Supplement: S1 Appendix — (PDF) [file pone.0176101.s001.pdf]

S1 Appendix. Limit behavior of the diffusion term. Here we show that the model approaches the diffusion equation in the continuum limit (eq. (1)) (see also [29]).

For generality, we consider the two-dimensional case. The displacement of individuals follows a discrete probability distribution $P^n_{x_{ij}}$ that an individual occupies a position x_{ij} at time t_n. Indices i and j represent discrete x and y coordinates respectively. $P^n_{x_{ij}}$ is discrete in time and space with $x_{(i+1)j} - x_{ij} = x_{i(j+1)} - x_{ij} = \Delta x$ and $t_{n+1} - t_n = \tau$. The probability P^{n+1}_r that the individual occupies the site $r = x_{ij}$ at time t_{n+1} depends only on the position of the individual at time point t_n, i.e., the model is a Markov process. The discrete-time Master equation describes the evolution of P^{n+1}_r:

$$P^{n+1}_r = \sum_{q \in \mathcal{N}(r)} P^n_q \, W^n(r|q). \tag{17}$$

Here, $\mathcal{N}(r)$ is the von Neumann neighborhood of range one (the cell r itself and its nearest neighbors $n_{\text{neighbors}}$). $W^n(r|q)$ is the probability of moving from cell q to cell r at time t_n. The transition probability $W^n(r|q) = 0$ when r and q are not in each other's neighborhood, and $\sum_{q \in \mathcal{N}(r)} W^n(r|q) = 1$.

We rewrite eq. (17):

$$P^{n+1}_r = \sum_{q \in \mathcal{N}(r),\, q \neq r} P^n_q \, W^n(r|q) + P^n_r \, W^n(r|r) \tag{18}$$

and solve for $W^n(r|r)$ (the movement is symmetrical so that $W^n(q|r) = W^n(r|q)$):

$$W^n(r|r) = 1 - \sum_{q \in \mathcal{N}(r),\, q \neq r} W^n(q|r). \tag{19}$$

The difference $P^{n+1}_r - P^n_r$ is derived by combining eq.(18) with eq.(19):

$$P^{n+1}_r - P^n_r = \sum_{q \in \mathcal{N}(r),\, q \neq r} P^n_q \, W^n(r|q) - P^n_r \sum_{q \in \mathcal{N}(r),\, q \neq r} W^n(q|r). \tag{20}$$

For the simultaneous random walk the transition probabilities are:

$$W^n(r|q) = P_{\text{move}} \frac{1}{n_{\text{neighbors}}} \quad \text{for} \;\; \text{q} \neq \text{r} \tag{21}$$

$$W^n(r|q) = P_{\text{stay}} = 1 - P_{\text{move}} \quad \text{for} \;\; \text{q} = \text{r}\ . \tag{22}$$

Using this definition of $W^n(r|q)$, equation (20) becomes:

$$P_r^{n+1} - P_r^n = \frac{1}{n_{\text{neighbors}}} P_{\text{move}} \left(\sum_{q \in \mathcal{N}(r), q \neq r} P_q^n - n_{\text{neighbors}} \cdot P_r^n \right). \tag{23}$$

If the total number of individuals is N_0, then the density of the individuals is $\rho = N_0 P_r^n / \Delta x^2$. Equation (23) then yields the finite-difference approximation of the diffusion equation (1) and approaches (1) in the continuum limit ($\tau \to 0$, $\Delta x \to 0$). The resulting diffusion coefficient is (eq. (3)

$$D = P_{\text{move}} \frac{1}{n_{\text{neighbors}}} \frac{(\Delta x)^2}{\tau}. \tag{24}$$
