# Supplementary material for: Individual-based modelling of population growth and diffusion in discrete time
Source: PLoS One. 2017 Apr 20;12(4):e0176101. doi: 10.1371/journal.pone.0176101 (PMC5398609; doi:10.1371/journal.pone.0176101)
Supplement: S2 Appendix — (PDF) [file pone.0176101.s002.pdf]

S2 Appendix. Limit behavior of the growth term. We analyze the discrete-time Master equation to show that the IBM approaches the deterministic logistic equation in the continuum limit with $\tau \to 0$ and $K \to \infty$, holding N/K, β/τ, and δ/τ constant. First, we derive $\langle\Delta\rangle$ from eq. (7) [2]:

$$\begin{aligned}\sum_{N=0}^{\infty} N\left[P_N(t+\tau)-P_N(t)\right] = &\sum_{N=0}^{\infty} N \sum_{\Delta=1}^{N} T(N|N-\Delta)P_{N-\Delta} \\ &+ \sum_{N=0}^{\infty} N \sum_{\Delta=1}^{\infty} T(N|N+\Delta)P_{N+\Delta} \\ &- \sum_{N=0}^{\infty} N \sum_{\Delta=1}^{N} T(N+\Delta|N)P_{N} \\ &- \sum_{N=0}^{\infty} N \sum_{\Delta=1}^{N} T(N-\Delta|N)P_{N}. \end{aligned} \quad (25)$$

where the transition probabilities are given by

$$T(N+\Delta|N) = \sum_{i=\Delta}^{N} \binom{N}{i}\binom{N}{i-\Delta}\beta^i(1-\beta)^{N-i}\delta^{i-\Delta}(1-\delta)^{N-(i-\Delta)} \quad (26)$$

$$T(N-\Delta|N) = \sum_{i=\Delta}^{N} \binom{N}{i}\binom{N}{i-\Delta}\delta^i(1-\delta)^{N-i}\beta^{i-\Delta}(1-\beta)^{N-(i-\Delta)} \quad (27)$$

$$T(N|N) = \sum_{i=0}^{N} \binom{N}{i}\binom{N}{i}\beta^i(1-\beta)^{N-i}\delta^i(1-\delta)^{N-i}\ . \quad (28)$$

By shifting the variable N in the first two terms on the r.h.s. of eq. (25) by $+\Delta$ and $-\Delta$, and accounting for boundary conditions of transition probabilities, i.e., $T(-N|0) = 0$ and $T(N-\Delta|N) = 0$ for $\Delta > N$, we get

$$\langle N(t+\tau)\rangle - \langle N(t)\rangle = \sum_{N=0}^{\infty}\sum_{\Delta=1}^{N} \Delta \cdot T(N+\Delta|N)P_N - \sum_{N=0}^{\infty}\sum_{\Delta=1}^{N} \Delta \cdot T(N-\Delta|N)P_N. \quad (29)$$

Note, that the limit $N \to \infty$ of eq. (29) is the Forward Euler integrator.

We now examine the transition probabilities $T(N+\Delta|N)$ and $T(N-\Delta|N)$ in the limit $\tau \to 0$; hence $\beta \to 0$ and $\delta \to 0$. Expanding to lowest order around $\beta = 0$ and $\delta = 0$, the transition probabilities become

$$T(N+\Delta|N) \approx \binom{N}{\Delta}\beta^{\Delta}; \quad T(N-\Delta|N) \approx \binom{N}{\Delta}\delta^{\Delta}. \quad (30)$$

For small τ, the change in the number of individuals (see eq. (29)) is

$$\langle \Delta \rangle = \sum_{N=0}^{\infty} \sum_{\Delta=1}^{N} \Delta \binom{N}{\Delta} \beta^{\Delta} P_N - \sum_{N=0}^{\infty} \sum_{\Delta=1}^{N} \Delta \binom{N}{\Delta} \delta^{\Delta} P_N. \tag{31}$$

Again, we omit higher-order terms and get the standard formulation for a one-step birth-death process [2]

$$\langle \Delta \rangle = \sum_{N=0}^{\infty} N \beta P_N - \sum_{N=0}^{\infty} N \delta P_N = \langle N\beta \rangle - \langle N\delta \rangle . \tag{32}$$

In the limit $\beta, \delta, \tau \to 0$, we have

$$\left\langle \frac{\mathrm{d}N}{\mathrm{d}t} \right\rangle = \frac{(b_0 - d_0)}{\tau} \langle N \rangle - \frac{(b_0 - d_0)}{\tau} \frac{\langle N^2 \rangle}{K}. \tag{33}$$

In the zero-noise limit where $\mathrm{Var}(N) = \langle N^2 \rangle - \langle N \rangle^2 = 0$, equation (33) is the deterministic logistic growth (eq. (4)) with the growth rate $r = (b_0 - d_0)/\tau$.

This convergence is slow as some higher-order transition probabilities (eqs. (26, 27, 28)) have large binomial coefficients. Furthermore, when K increases, birth and death probabilities must decrease to ensure at most one birth or death event at a time step. More formally, we analyzed the error of the linear approximation of $T(N|N) \approx N \cdot (\beta + \delta)$. The linear approximation can be applied only when the relation $N\sqrt{\beta\delta} \ll 1$ is satisfied, e.g., $K \sim N = 10$ requires $\beta = \delta = \theta \ll 0.1$; $K \sim N = 500$ requires $\beta = \delta = \theta \ll 0.002$.
